# Aquatic Debris Detection Using Embedded Camera Sensors

**DOI:** 10.3390/s150203116

**Published:** 2015-01-30

**Authors:** Yong Wang, Dianhong Wang, Qian Lu, Dapeng Luo, Wu Fang

**Affiliations:** 1 Faculty of Mechanical and Electronic Information, China University of Geosciences, Wuhan 430074, China; E-Mails: wangdh@cug.edu.cn (D.W.); luodapeng@cug.edu.cn (D.L.); swmyang@126.com (W.F.); 2 Department of Geological Science and Engineering, Wuhan University of Engineering Sciences, Wuhan 430200, China; E-Mail: luqian8710@163.com

**Keywords:** aquatic monitoring, camera sensors, object detection, compressive sensing

## Abstract

Aquatic debris monitoring is of great importance to human health, aquatic habitats and water transport. In this paper, we first introduce the prototype of an aquatic sensor node equipped with an embedded camera sensor. Based on this sensing platform, we propose a fast and accurate debris detection algorithm. Our method is specifically designed based on compressive sensing theory to give full consideration to the unique challenges in aquatic environments, such as waves, swaying reflections, and tight energy budget. To upload debris images, we use an efficient sparse recovery algorithm in which only a few linear measurements need to be transmitted for image reconstruction. Besides, we implement the host software and test the debris detection algorithm on realistically deployed aquatic sensor nodes. The experimental results demonstrate that our approach is reliable and feasible for debris detection using camera sensors in aquatic environments.

## 1. Introduction

Water resources and aquatic ecosystems such as oceans, lakes, rivers and drinking water reservoirs are facing severe threats from floating debris. The majority of the debris comes from the human-created waste, which poses numerous risks to public health, ecosystem sustainability and water transport [1]. For instance, debris leads to fish deaths [2] and severe damage to fishing vessels [3]. It is of great importance to monitor aquatic debris and take preventive measures for the potential risks. In the past few decades, debris monitoring has primarily been conducted by manual spotting using patrol boats. However, this solution is labor-intensive and only viable for small-scale monitoring. In particular, due to the periodic nature of these inspections, they are prone to miss sudden events like the debris washed down by a heavy rain. More advanced autonomous underwater vehicles (AUVs) and robotic platforms [4,5] have been previously utilized to sense aquatic environments. However, the limiting factors such as high manufacturing costs, high weight and large size, greatly restrict their deployment over larger areas for spatially and temporally monitoring scattered debris objects. Nowadays remote sensing is used as an alternative approach for large-scale aquatic debris monitoring. In [6], a balloon equipped with a digital camera is used to monitor marine and beach litter. Nevertheless this approach is only effective for one-off and short-term monitoring of highly concentrated debris fields. The work in [7] proposed satellite imaging to detect accumulation areas of debris in the open ocean. However, debris monitoring based on this method has a low resolution both in time and space because of the high operational cost.

**Figure 1.**
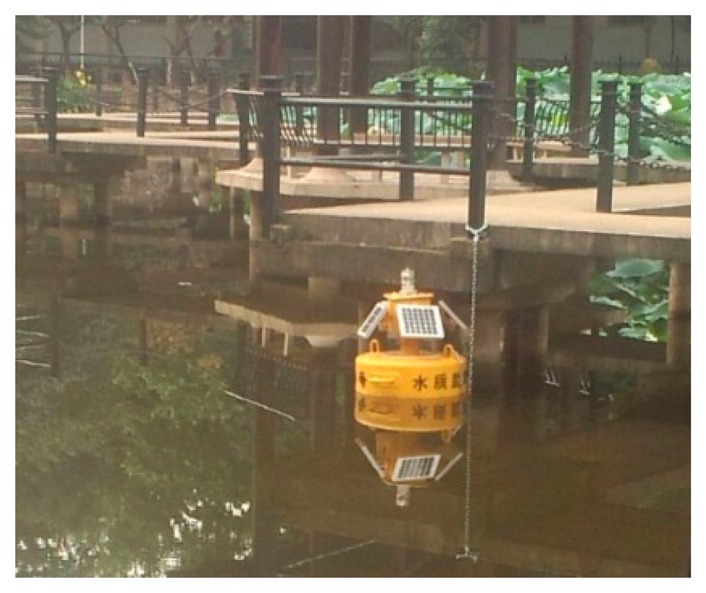
A prototype of aquatic sensor node.

Recently, there is an increasing interest in the development of autonomous, *in situ*, real-time sensing platforms for aquatic environments [8–10]. Moreover, sensing platforms equipped with embedded camera sensors provide a promising solution for efficiently and intuitively observing aquatic environments, so we have developed an inexpensive, multipurpose aquatic sensor node enabling long-term aquatic monitoring. Figure 1 shows a completed aquatic sensor node working in a pond.

The node includes several kinds of aquatic sensors submerged in water and an onboard camera sensor on top. The former periodically collect the water-quality data and the latter captures images for debris detection. However, there are some specific problems awaiting solutions for the vision-based aquatic debris detection. First, due to the impact of uncontrollable water surface disturbances such as waves, camera shaking and swaying reflections on the water, it is quite difficult to reliably identify debris objects. Second, embedded platforms are not competent for continuous and real-time image processing because of the constraints on computational power and memory [11]. This means the debris detection algorithm should have low computation complexity whilst achieving desirable detection accuracy. Third, due to the restricted accessibility for replacing batteries of aquatic sensor nodes, the limited energy supply remains a challenge for long-term monitoring. Apart from the acquisition, processing and transmission of water-quality data, both debris detection and image transmission incur high energy consumption. Though node energy can be replenished continuously with energy harvesting from solar and other sources, the amount of energy available is highly dependent on the geographic location, season, and deployment environment. Thus the key idea is still to take some effective measures to decrease the energy consumption.

Due to space constraints, this paper is restricted to discuss the vision-based debris detection using our embedded sensing platform. The main contributions of this paper are as follows:

(1) We propose an accurate and computationally efficient approach to debris detection. On the basis of both compressive sensing and adaptive block size, our method reduces the dimensionality of the image while retaining most of helpful information, more importantly, achieves different granularities of debris detection according to variations of the aquatic environment.
(2) We demonstrate that the proposed method can effectively mitigate the impact of disturbances and reliably detect debris objects. Moreover, we validate the efficiency in terms of detection reliability and average runtime through extensive experiments and comparisons.
(3) We present the design of aquatic sensor nodes and implement the debris detection algorithm on the embedded platform. The realistic deployment shows our approach consumes less energy consumption and hardware resources. Moreover, debris images can be uploaded through the host software, and the compressive transmission has a low communication overhead and latency.

The remainder of this paper is organized as follows: Section 2 reviews the related works. Section 3 provides the specifics and rationale of aquatic sensor nodes. Section 4 presents the debris detection algorithm and the compressive transmission of debris images. Section 5 discusses the results of extensive experiments and realistic implementation on the sensing platform. Section 6 concludes this paper.

## 2. Related Works

Traditionally, aquatic monitoring based on sensor networks has focused on low-level sensors that measure 1D data signals, e.g., dissolved oxygen, conductivity, and temperature, which limit the ability to provide richer descriptions of aquatic environments. Recent developments in wireless sensor networks and distributed processing have made the use of camera sensors in environmental monitoring possible [8,12]. Several low-power wireless sensing platforms integrating camera sensors have been investigated. Cyclops [13] integrates a CMOS camera module hosted by a MICA2 mote. It can perform simple image processing like background subtraction using frame difference. CITRIC [14] consists of a camera daughter board connected to a TelosB mote. The platform has been successfully applied to several typical applications, e.g., object detection and recognition. Besides, multi-tier sensor networks seek to provide a low-latency yet energy-efficient camera sensing solution. SensEye [15] is a notable example which consists of low-power and low-resolution cameras at the bottom tier that trigger higher resolution cameras at the upper tier in an on-demand manner. The study in [10] presents a smart phone-based sensing platform that utilizes the built-in camera, inertial sensor, and other resources. Different from aforementioned sensing platforms, we aim to combine camera sensors with other types of aquatic sensors into a sensor node for multipurpose aquatic monitoring.

**Figure 2.**
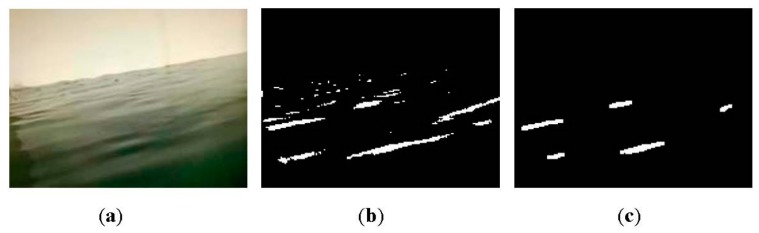
An example of pixel-based detection. (**a**) Original frame; (**b**) Foreground extraction; (**c**) The result with morphological operations.

The key to aquatic debris detection is to extract the foreground objects from video sequences captured by camera sensors. A common way to foreground extraction is background subtraction. One such means, Gaussian mixture model (GMM) [16] has been widely adopted because of its ability to deal with subtle illumination changes. Several modified methods involving the number of Gaussians, learning rate and parameters update [17–20] also have been proposed. However, this kind of method is computationally intensive and subject to non-static scenes. A non-parametric method called kernel density estimation (KDE) has been proposed for background modeling [21], but this method has high memory consumption. In our earlier work [22], foreground pixels are identified by estimating the anomaly probability of each pixel, which outperforms GMM and KDE in coping with scenes including small motions and shadows. Recently, an efficient background subtraction technique called ViBe has been presented in [23]. It is reported that this method is superior to proven state-of-the-art methods in terms of both computation speed and detection rate. An example of debris detection using ViBe is shown in Figure 2.

From the results, large numbers of pixels corresponding to heavy waves are falsely identified as debris objects. Though ViBe outperforms mainstream techniques, it still has difficulty differentiating between foreground and complex background disturbances. It is concluded that the pixel-based approaches cannot be readily applied to background subtraction in dynamic water environments. Moreover, it is noteworthy that image processing pixel by pixel often incurs significant computation overhead, this makes many existing background subtraction algorithms unsuitable for embedded platforms due to the resource constraints. As is well known, compressive sensing (CS) is capable of significantly reducing the sampling rate and data dimension while retaining much of the information [24]. In order to realize computational efficiency, the work in [25] uses CS to reduce the dimensionality of the image and then applies GMM to the reduced dimension data for background subtraction. As mentioned earlier, this approach models the background using GMM, which also faces unique challenges in aquatic environments. Besides, an approach based on CS performs background subtraction by learning and adapting a low-dimensional compressed representation of the background [26]. The limitation lies in the fact that objects in the foreground need to occupy only a small portion of the camera view in order to be detected correctly. In this paper, we lay stress on a computationally efficient and accurate background subtraction algorithm based on CS for real-time aquatic debris detection.

## 3. Overview of Aquatic Sensor Nodes

### 3.1. Aquatic Sensor Node

**Figure 3.**
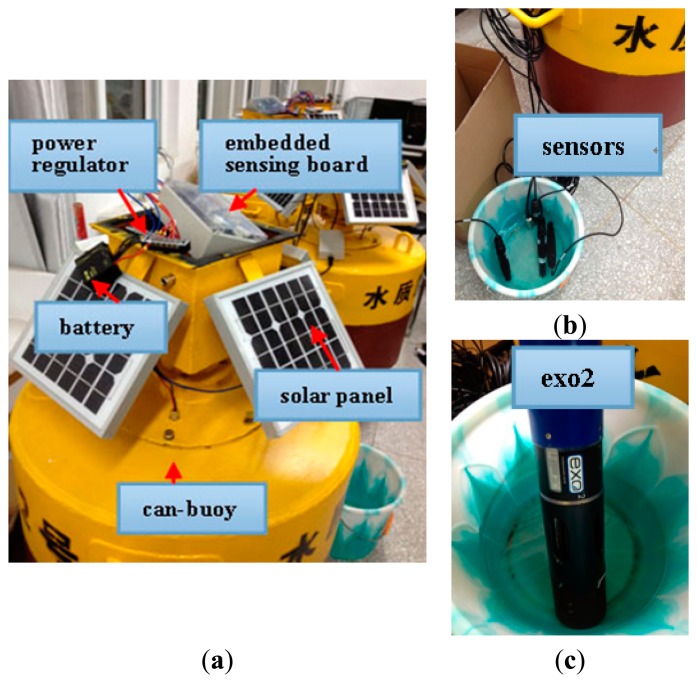
Components of an aquatic sensor node. (**a**) The functional modules; (**b**) Aquatic sensors; (**c**) The sonde EXO2.

We develop a heterogeneous sensor platform for comprehensive aquatic environments monitoring. Figure 3a shows the components of the aquatic sensor node. The main body is a can-buoy, as the carrier of the sensing platform, which is deployed in monitoring waters. Embedded sensing board is the hardcore of the aquatic sensor node, and responsible for signal sensing, processing and communication. According to the desired application, the sensor node is also equipped with five types of aquatic sensors, namely, depth sensor, temperature sensor, conductivity sensor, dissolved oxygen sensor, and pH sensor, as shown in Figure 3b. All of them connect by sensor interfaces with the embedded sensing board. The measurements with low-end sensors usually deviate from actual values, as a result, we use EXO2, a multiparameter water quality sonde from YSI shown in Figure 3c, to crosscheck the calibration of these sensors.

It should be noted that long-term aquatic monitoring is power-hungry, especially real-time image processing and wireless communication. Therefore, energy harvesting from the surrounding environment must be considered when designing aquatic sensor nodes. As shown in Figure 3a, the battery contains five rechargeable 4.2V 3200 mAh lithium cells which are connected in parallel, that is, the total battery capacity is 16,000 mAh. Four solar panels connected in parallel whose maximum output voltage is 17.5 V are capable of supplying up to 1 A of current to charge the batteries. Thus the functionality of the power regulator is two-fold. First, it protects the batteries from overcharge or discharge under software control. Second, it serves as DC-DC converter which reduces the voltage of solar panels down to the required voltage for the battery. Tests indicate that the entire current consumption including all sensors and associated peripherals approximates 530 mA. If the node operates continuously, on average, this battery has a lifetime of about 30 h. Note that the battery lifetime can be increased by enabling power management, such as duty cycling, trigger mode and shutting down specific sensing modules.

### 3.2. Embedded Sensing Board

**Figure 4.**
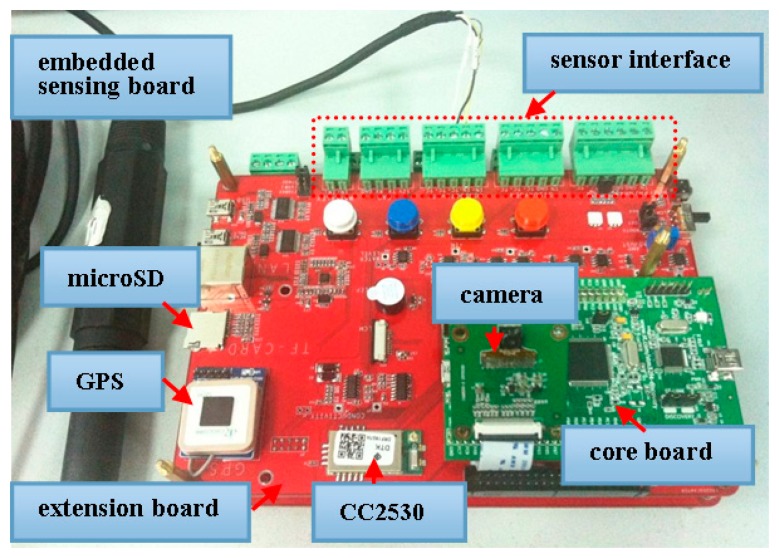
Embedded sensing board.

Figure 4 shows the embedded sensing board of a small size 9 cm × 12 cm, which consists of a core board and an additional extension board. The core board comprises a 32-bit 180 MHz Cortex-M4 processor which meets the low power requirements, a detachable camera sensor, and an external SDRAM of 64 Mbits. The processor embeds the digital camera interface that can connect with a camera to receive video data. The camera adopts OV9655, a low voltage CMOS image sensor that provides the full functionality of a camera and image processor on a single chip. The SDRAM is for storing image frames during debris detection, it can store 27 frames raw RGB images with a CIF resolution (3 bytes/pixel × 352 × 288 × 27 frames = 8 MB), which is more sufficient than the requirement for background subtraction. On the other hand, the bottom expansion board contains ZigBee module (CC2530) for networking and wireless communication, GPS for localization, and a 4 GB microSD card for local bulk data storage in case of intermittent network connection or network congestion. In addition, the expansion board has sensor interfaces which allow aquatic sensors to connect with the processor, and other peripherals. In short, the design uses a small number of functional blocks to minimize size, power consumption and manufacturing cost.

The basic principle of our sensing platform is introduced briefly as follows. Each sensor node can be configured as a coordinator or an end-device, and multiple nodes can be deployed to form a star or tree network through onboard CC2530. All of these become quite easy using Texas Instruments Z-Stack [27], a ZigBee compliant protocol stack for IEEE 802.15.4, run on the CC2530. The coordinator acting as the host node that is connected to a PC sends user commands to the sensor network and receives data packets from sensor nodes. For slave sensor nodes, the CC2530 receives commands from the host node and instructs the processor to execute different tasks. The signals of aquatic sensors, after conditioned by the sensor interface circuits, are sent to the analog-to-digital converter port of the processor. Furthermore, the processor controls the camera sensor to capture images and then performs debris detection. Both the sensor data and the debris images are sent back to the host node via the CC2530. Note that the processor will shut down the corresponding function module to minimize power consumption when a certain monitoring task is not needed.

## 4. Compressive Detection Approach

### 4.1. Compressive Sensing

Compressed sensing theory [24,28] asserts that a sparse signal can be usually sampled by a signal-independent random projection and reconstructed from far fewer measurements than suggested by the Nyquist sampling theory. Obviously, it is well adapted for fast, efficient and in-expensive signal processing algorithms and embedded devices. The key idea behind CS is that, if a signal ***x*** in the high dimensional space is sparse, and then it can be projected into a much lower dimensional signal ***y*** without losing much information. Therefore, instead of the original signal ***x***, we can work on the linear measurements ***y***, so as to achieve computational efficiency as well as accuracy. In order to explain the rationale, we consider an image ***X*** of size *H* × *W* and then convert it into a column vector ***x*** of size *N* × 1 (*N* = *H* × *W*) by concatenating the individual columns of ***X*** in order. The linear random projections of the image vector ***x*** are measured as follows:

(1) y=Φx

where ***y*** contains *M* measurements and ***Φ*** is an *M* × *N* measurement matrix where *M* ≪ *N*. For example, we can obtain the linear measurements of size 16 × 1 by applying a 16 × 1024 measurement matrix to the image vector of size 1024 × 1. The strength lies in the fact that the data dimension to be processed is markedly reduced.

Note that the recovery of image vector ***x*** from the measurements ***y*** is underdetermined because *M* is much smaller than *N*. However, the two additional assumptions make recovery possible [29]. First, ***x*** exhibits sparsity in some domain. Assume that ***x*** can be expressed as ***x*** = ***Ψθ***, where ***Ψ*** denotes the sparsity basis in a transform domain and ***θ*** is an *N* × 1 coefficient vector over ***Ψ***. ***x*** is said to be sparse in ***Ψ*** if the vast majority of elements in ***θ*** are either zeros or very close to zeros. Second, the measurement matrix ***Φ*** is required to obey the restricted isometry property (RIP) [24]. This means ***Φ*** is incoherent with ***Ψ***. Surprisingly, incoherence holds with high probability between a sparsity basis and a randomly generated matrix, e.g., Gaussian, Bernoulli and Toeplitz [26]. In this case, it is possible to recover ***x*** with high probability based on the following ℓ_1_ norm optimization problem.

(2) $$\begin{array}{cc} \hat{x}=\arg\min{\left\|x\right\|}_{1} & \text{s.t.}y=\Phi x \end{array}$$

### 4.2. Debris Detection

**Figure 5.**
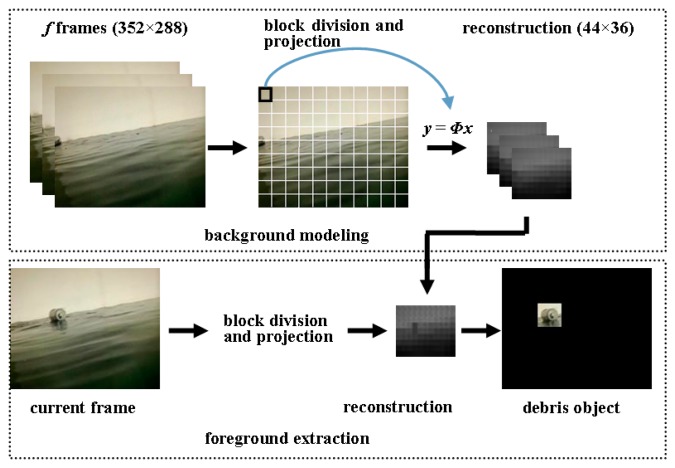
Compressive detection of aquatic debris.

Figure 5 illustrates the outline of the proposed debris detection algorithm. In the phase of background modeling, the image is divided into sub-blocks whose size can be arranged to suit monitoring requirements. For each block, the linear measurements are computed by using Equation (1). Then a new image is reconstructed according to the measurements of all blocks, which contains useful information of the original image. For example, as shown in Figure 5, we break a 352 × 288 image into smaller blocks of 32 × 32 pixels. Each block can be expressed as a 1024 × 1 vector. After the projection with a 16 × 1024 measurement matrix, the high dimensional vector is transformed into a 16 × 1 vector. We can see that a 32 × 32 image block is replaced with another block of size 4 × 4, that is, the 352 × 288 original image can be characterized by the 44 × 36 reconstructed image. It is noteworthy that the background model is initialized from *f* reconstructed frames. In the phase of foreground extraction, current frame is processed in the same way as block division and projection. Each pixel in the reconstructed frame is compared to background samples to determine if it is classified as foreground. We detail the debris detection algorithm and the related issues in the following sections.

#### 4.2.1. Block Projection and Image Reconstruction

HSV (*hue*, *saturation*, and *value*) representation is robust to illumination changes and more effective than RGB in the presence of reflection in water environments [10]. In view of this fact, the video frames are first converted from RGB to a HSV model. To reduce computation overhead in the image processing, in our approach, only the *value* component in HSV that indicates the lightness of the color is chosen for foreground extraction.

Prior to reducing the dimensionality by random projections, the image should be divided into some sub-blocks. We experimented with different block sizes and found that the differences in detection results are really noticeable, especially in complex water environments. Therefore, instead of a fixed block of pixels like the work in [25], we adopt an adaptive strategy where the block size is flexible and can be set at 8 × 8, 16 × 16 and 32 × 32. The choice of the block size conforms to the following criteria: first, the stronger the environment disturbance, the larger the block size. Environment disturbances include camera shaking, waves and swaying water reflection, *etc.*, all of which result in sudden changes of pixel values in consecutive frames. Thus the environment disturbance can be estimated through frame difference. We count the pixels that have great change and compute the ratio of these pixels to the image pixels. Obviously, the ratio increases with the strength of the environment disturbance. The default block size in our method is set 16 × 16. When the ratio is less than 10%, the block size is set 8 × 8. Conversely, the block size is set 32 × 32 when the ratio exceeds 20%. Second, the block size depends on the requirement for the detection reliability, *i.e.*, the size of debris object and detection granularity. As we know, the random projections of image block will weaken object features and lose some image information. If the block size is overlarge, small debris objects will not be detected. Meanwhile, the detection granularity, including debris position and area, will decrease with increasing the block size, as described in Section 5.1.

Subsequently, the pixel values of each block can form a high dimensional vector ***x*** of size *N* × 1, and then it is compressed to the *M* × 1 vector ***y*** in which the elements are far fewer than that of the block. The measurements, instead of the original data, are used to decide whether there exist foreground objects or not. As a matter of fact, 16 measurements per block are enough to achieve good performance for foreground extraction because the larger values of *M* give similar results. We therefore assume *M* = 16 throughout this paper. Furthermore, measurement matrices randomly generated by a Bernoulli distribution of 0's and 1's, which satisfy RIP, are regarded as a good choice for preserving the information of the image block in the measurements. Note that once a measurement matrix is generated in the beginning, the same matrix is used for each block during the debris detection.

**Figure 6.**
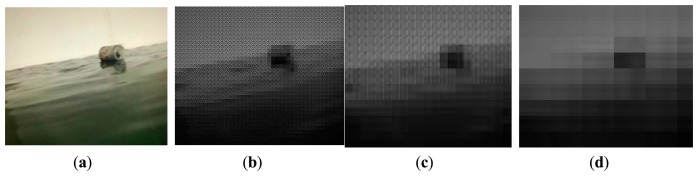
Image reconstruction under different block sizes. (**a**) Original frame of size 352 × 288; (**b**–**d**) denote reconstruction frames with the block of size 8 × 8, 16 × 16, 32× 32, respectively.

To reconstruct the new image, the 16 measurements corresponding to a block are transformed into a new block of size 4 × 4. Figure 6 gives the results of image construction under different block sizes. Take Figure 6b for example, where each block of size 8 × 8 is converted to another block of size 4 × 4. The new image is a quarter of the size of the original image. This allows us to work with lower dimension data, e.g., 176 × 144 pixels instead of 352 × 288. Moreover, the sizes of the new images, as shown in Figure 6c,d, fall from 352 × 288 to 88 × 72 and 44 × 36 respectively, but the reconstruction frames still preserve adequate object features. It can be expected that the block projection and image reconstruction result in computational efficiency with little loss of helpful information.

#### 4.2.2. Background Modeling

In our solution, we build a model with real observed pixel values from a sequence of frames. The underlying assumption is that the observed values have a higher probability of being observed again than values not yet encountered, which is reasonable from a stochastic point of view. From Figure 5, *f* frames at the beginning of video are reconstructed in sequence, such that background modeling can be executed on the *f* new images. If the block size is chosen as 16 × 16, a video sequence of size 352 × 288 can be modeled through the *f* reconstruction frames of size 88 × 72. This means the computation efficiency can be improved greatly because the number of pixels need to be modeled is reduced. For each pixel, no extra computations, we just need to pick *f* values of the pixel from the *f* reconstruction frames, and the pixel is modeled by the collection of sample values. Thus the background model of pixel (*i*, *j*) can be described as:

(3) $$\text{M}(i,j)=\left\{{\text{v}}_{1}(i,j),{\text{v}}_{2}(i,j),\cdots {\text{v}}_{f}(i,j)\right\}$$

where v*_s_*(*i*, *j*) (*s*=1, …, *f*) denotes the *value* component of pixel (*i*, *j*) in the *s*-th reconstruction frame. Taking into account the impact of environment disturbances, *f* needs to be large enough to guarantee the diversity of background samples. From our experiments, this strategy has proved to be successful under *f* = 15.

We now formulate briefly the memory requirement of the background model. As described before, each pixel is represented by its *value* component in HSV (1 byte). The required memory includes two parts below. First, a storage space is allocated for a frame because the original images are processed in sequence, which is 99 KB (1 byte/pixel × 352 × 288). Second, the reconstruction frames occupy 93 KB (1 byte/pixel × 88 × 72 × 15 frames) memory. The total memory is 192 KB, which is considerably lower than the memory the sensing platform can afford.

#### 4.2.3. Foreground Extraction

After background modeling, the next frame is first reconstructed by block division and projection, as shown in Figure 5. For the reconstructed image, we need to identify which pixels belong to background according to the background model. In our solution, a *value* matching-based method is adopted to classify each pixel in the reconstruction frame. Since the operations to be carried out on each pixel are identical, we describe the operations on a pixel. For the pixel (*i*, *j*), we compare its v(*i*, *j*) to the samples in **M**(*i*, *j*), if the absolute difference between them is less than a given threshold, *Th*, then v*_k_*(*i*, *j*) can be seen as a match for the pixel. When the number of the matches exceeds the threshold *τ*, the pixel should be marked as background. Formally, a background pixel satisfies the following condition:

(4) $$\#\left\{\begin{array}{ll} \left|\text{v}(i,j)-{\text{v}}_{k}(i,j)\right|<Th, & {\text{v}}_{k}(i,j)\in \text{M}(i,j) \end{array}\right\}>\tau$$

As can be seen, the classification of a pixel only involves simple subtraction operations, which is computationally inexpensive and time-saving. The classification accuracy of a pixel depends on the two thresholds. Experiments show that *Th* = 25 and *τ* = 3 are appropriate for a great variety of practical applications.

**Figure 7.**
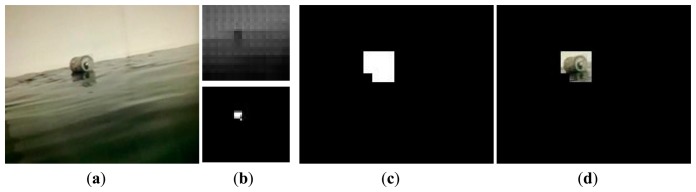
Foreground extraction with 16 × 16 block. (**a**) Original frame; (**b**) The reconstruction frame and its foreground mask; (**c**) The real foreground mask with scale = 4 and bicubic interpolation; and (**d**) Foreground object in original image.

Figure 7 illustrates the procedure of the foreground extraction. First, the original image is reconstructed and the corresponding foreground mask is determined according to Equation (4), where foreground and background pixels are labeled as white and black respectively.

In order to extract the foreground objects, the next step is to recover the size of the foreground mask referring to the original image. In view of the block size is 16 × 16, the real foreground mask is four times the size of the obtained foreground mask, which can be easily implemented by image scaling interpolation. Finally, the debris object can be extracted from the original image according to the foreground area.

#### 4.2.4. Background Model Update

To achieve accurate debris detection over time, it is crucial to update the background model. As described in ViBe [23], older samples in the background model should not be compulsorily discarded or replaced by the new ones, it is more appropriate to ensure a monotonic decay for the remaining lifespan of these samples. Besides, in many practical situations, it is not necessary to update each background pixel model for each new frame. When a pixel is classified as the background, a random probability determines whether this pixel is used to update the corresponding pixel model. Similar to this idea, our update scheme is described as follows. Assume that the background model at time *t* is **M***_t_* = {**M***_t_*(*i*, *j*)}, and the probability of a sample in the model being preserved after the update of the pixel model is given by (*f* − 1)/*f*. For a reconstructed frame, as shown in Figure 7b, its foreground mask can be determined by matching **M***_t_*. If a pixel (*i*, *j*) is marked as background, its value is likely to be incorporated into the background model at time *t* + 1, **M***_t_*_+ 1_(*i*, *j*). This means any sample in **M***_t_*(*i*, *j*) will be chosen with probability of 1/*f* and replaced by the current pixel with a probability of 1/*δ*. In the aquatic monitoring, the value of *δ* between 5 and 10 is suitable. A smaller threshold should be applied if the environmental change is more severe. This strategy greatly speeds up the background model update without reducing detection accuracy.

On the other hand, if the pixel (*i*, *j*) is classified as foreground, its background model at time *t* + 1 will remain the same as that at time *t*, that is, the pixel value is never incorporated into the background model. However, a progressive incorporation of foreground samples in the background model should be considered. For example, when a foreground object starts moving, the ghost area must eventually become part of the background after a given time. To solve this problem, our solution is to count the number of consecutive times that a pixel at a particular location has been marked as foreground. If the number reaches a given threshold (e.g., 50), the pixel at this location is labeled as background. Then the current pixel value will randomly substitute for a sample in the background model.

### 4.3. Compressive Transmission

The debris images allow us to study fine-grained spatial distribution and temporal movement of floating debris. However, several factors seriously impede this function [30]. The limited ZigBee bandwidth, only up to 250 kbps, cannot satisfy the needs of real-time image transmission. Moreover, the limited power supply in sensor nodes becomes the bottleneck in transmitting images. Direct transmission of image data will quickly exhaust sensor nodes' energy. Thus the key is how to represent the image using as little data as possible and in turn recover the image from these data without severe image distortion. Through doing this, image transmission can be cast as a sparse signal recovery problem.

In the CS theory, a signal can be recovered from a small set of distinct measurements, provided that the signal is sparse or compressible in original or some transform domain. Remarkably, the debris images obtained by foreground extraction are sparse. As shown in Figure 7d, only a small part of the pixels in the debris image have nonzero values. As the image encoder, what the slave sensor node has to do is to obtain the linear measurements of the debris image by using simple random projections. Intuitively, the way of only transmitting the measurements is severely desirable for the resource-deprived embedded sensing platform. In turn, the powerful user terminal (e.g., PC) functioning as the decoder is capable of recovering the debris image.

To reduce communication and computation overhead, the measurements for image recovery should be as few as possible. Number of measurements needed for exact image reconstruction depends on particular recovery algorithm being used. Considering the fact that nonzero coefficients are not randomly distributed but clustered spatially in the foreground extraction image, the authors of [31] propose a new greedy sparse recovery algorithm called dynamic group sparsity (DGS). This method considers both sparsity and group clustering priors rather than only sparsity as in traditional recovery algorithms, which enables stable recovery with less measurement requirement and lower computation complexity. More details of DGS are available in [31] and will not be described here due to space limitations. In view of the advantages, we utilize DGS to solve the image recovery in this paper.

To improve computational efficiency, the debris image is divided into four blocks of pixels. Each block is converted into a column vector ***x****_i_* (*i* =1, …, 4) of size *n* × *p*, where *n* denotes the product of the block height and width, *p* is the channel numbers of debris images. Then random projections are executed for each block using Equation (1). The projection matrix ***Φ*** is generated by creating an *m* × *n* matrix with i.i.d. draws of a Gaussian distribution *N* (0; 1), and then the rows of ***Φ*** are normalized to the unit magnitude. After this, we can obtain the measurements ***y****_i_* of size *m* × *p*, which contain most of the information in this block of pixels. In the second step, the host node receives ***y***= {***y****_i_*} and ***Φ*** from the slave sensor nodes and recovers debris images based on DGS. It is worth mentioning that the *k*-sparse signal ***x*** ∈ R*^n^*, *k* ≪ *n*, can be recovered by DGS using *m* = ***O*** (*k*) measurements, which is a significant improvement over *m* = ***O*** (*k*log(*n*/*k*)) that would be required by conventional CS recovery algorithms. To quantitatively evaluate the recovery performance, the normalized recovery error is defined to indicate the difference between the recovered signal ***x̂****_i_* and the ground-truth ***x****_i_*:

(5) $$\text{err}=\frac{\sum_{i}{\left\|{\overset{⌢}{x}}_{i}-x_{i}\right\|}_{2}}{\sum_{i}{\left\|x_{i}\right\|}_{2}}$$

**Figure 8.**
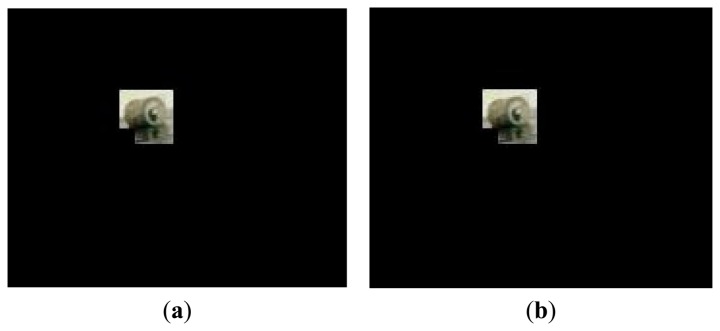
Image sparse representation and recovery. (**a**) Debris image; (**b**) Recovered image (*err* = 0.0151).

We find that the DGS achieves almost perfect recovery when the number of measurements is only two times of the sparsity of blocks. Figure 8 shows the recovery result of the debris image in Figure 7d. According to Equation (5), the normalized recovery error is 0.0151, which indicates that the recovery performance is good enough. In this experiment, each color channel of the debris image contains 4260 measurements. Each measurement occupies one byte, so the total linear measurements are 4260 × 3 bytes. Compared to the original debris image (352 × 288 × 3 bytes/pixel), the data needed to be transmitted is reduced by 95.8%, which is a very encouraging result. If we consider the gray image, instead of color image, two-thirds of measurements will be further compressed. Thus such a small quantity of measurements does not incur high communication overhead and latency in the wireless transmission.

## 5. Experimental Results

### 5.1. Effectiveness Analysis

**Figure 9.**
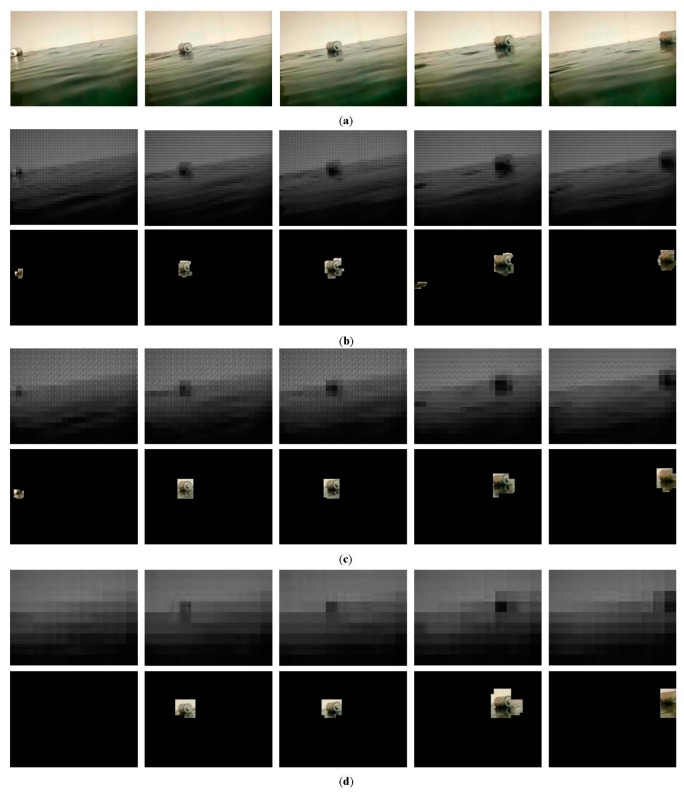
Debris detection under different block sizes. (**a**) Original frames 41, 82, 94, 119 and 139 from left to right; (**b**) 8 × 8 block size; (**c**) 16 × 16 block size; and (**d**) 32 × 32 block size.

We now describe the effectiveness and intrinsic properties of the proposed method, and adequately address the impact of the block size on the debris detection. The tests are conducted on a video sequence of 150 frames captured from an imitative aquatic environment in the laboratory. The image size is 352 × 288 and the frame rate is 25 fps. The parameters in this experiment are as follows: *M* = 16, *f* = 15, *Th* = 25, *τ* = 3, and *δ* = 10. Figure 9a is the original frame where a tin can is floating on the water in the presence of persistent waves. Figure 9b–d show the results of debris detection under 8 × 8, 16 × 16 and 32 × 32 block size, respectively. Specifically, the first row is the reconstructed frame, and the second row is the detected debris object. Note that all reconstructed frames are shown in the same size as original images in order to distinguish differences between them. As can be seen, the larger the block size, the more image detailed information is lost. And because of this, our method effectively mitigates the impact of waves. In general, under three different block size, the proposed method can almost identify the debris object. However, it is worth focusing on some differences and details. From the result of frame 119 shown in Figure 9b, we can see that a small piece of background corresponding to a heavy wave is incorrectly detected as foreground. This means that a small block size is not able to handle the severe disturbance. Also, the result of frame 41 shown in Figure 9d shows that no debris objects is detected. Foreground objects with small size are likely to be omitted due to the loss of image information under a large block size. Furthermore, it is clear that the debris object location and area can be more accurately pinpointed by using a small block size. The detection results in Figure 9d contain more background surrounding the debris object than those in Figure 9b. Comparatively, when we take into account both detection accuracy and granularity, the 16 × 16 block size is a better choice for this aquatic environment, as shown in Figure 9c.

As said above, the detection granularity can be improved by reducing the small block size. However, the improvement is at the cost of sacrificing computation efficiency. Experiments show that, when block size is set 8 × 8, 16 × 16 and 32 × 32, the average computation times per frame based on our embedded sensing platform are 426.84, 219.61 and 167.39 ms, respectively. The processing time of the 32 × 32 block size respectively reduces by about 60.8% and 23.8% compared with those of the two others. In summary, our approach allows the sensing platform to adjust adaptively the block size according to environment disturbance. For example, in this experiment, we find that the ratio of the pixels whose values have great change to the image pixels is 15.7%. As described in Section 4.2.1, the ratio means that the optimal value of block size is 16 × 16, which has been demonstrated experimentally in Figure 9.

If strong winds cause the heavier waves, the 32 × 32 block size is preferred. The golden rule of choosing the block size is to minimize the computation overhead while guaranteeing the detection granularity. Moreover, the computational complexity of our method fully satisfies the requirement for real-time debris monitoring. For instance, the processing speed is up to 5fps using 16 × 16 block size, which is fast enough for debris detection due to the fact that debris usually has a slow drifting speed.

### 5.2. Performance Evaluation

**Figure 10.**
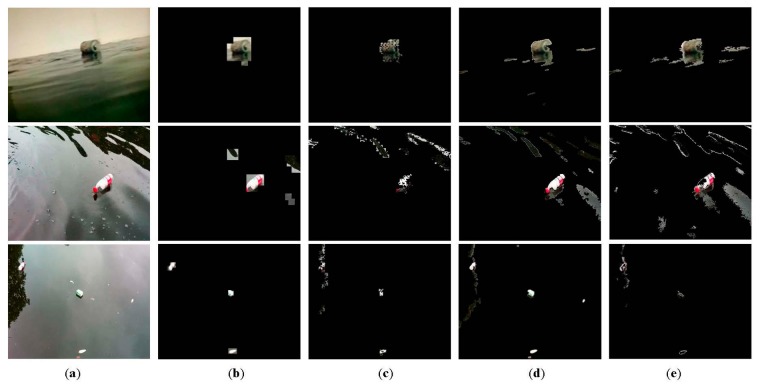
Comparative foreground extractions of four methods. (**a**) Original frames; (**b**) Our approach with 16 × 16 block size; (**c**) CS-MOG; (**d**) ViBe; and (**e**) GMM.

Three representative video sequences of 352 × 288 pixels are used to evaluate the performance of our algorithm. The first two sequences concern the debris detection under condition of waves, especially, heavy waves in the second, and the third concerns the water reflections of swaying trees. We compare our algorithm with three classical approaches: (1) CS-MOG [25]; (2) ViBe [23]; and (3) GMM [16] in which all of the parameters use the proposed default values. Figure 10 shows examples of foreground extraction for a certain frame of the three sequences, where debris objects are segmented from original images with four methods.

We can see from Figure 10d,e that pixel-based methods like ViBe and GMM, cannot deal with waves and swaying reflections, and a large number of background pixels are incorrectly classified as foreground. By contrast, CS-MOG uses the CS-based block processing, which can eliminate environment disturbances to some extent. However, the foreground extraction evidently deteriorates when there exist severe disturbance. As shown in Figure 10c, due to the fixed 8 × 8 block size, CS-MOG fails to identify debris objects accurately. In addition, this method applies GMM to eight projection values for background subtraction, which leads to many holes and fragments in the detected debris objects. Compared to the three methods, our approach gives consideration to both the debris detection and background suppression. Figure 10b illustrates that our approach accurately extracts the debris objects even if the block size is set 16 × 16. Three points are worth highlighting. First, due to using block division and random projection in our solution, part of background pixels surrounding the debris object are marked as foreground, which have no perceptible effect on debris detection. Second, the foreground extraction of the second sequence contains several background pixel blocks because of the effect of heavy waves. The reason for this is that we use the 16 × 16 block size, which is unsuitable for the aquatic environment. This problem can be easily solved by using the 32 × 32 block size. Third, an extremely low false alarm of our approach makes any post-processing unnecessary, which further alleviates the total computational cost of the debris detection.

**Figure 11.**
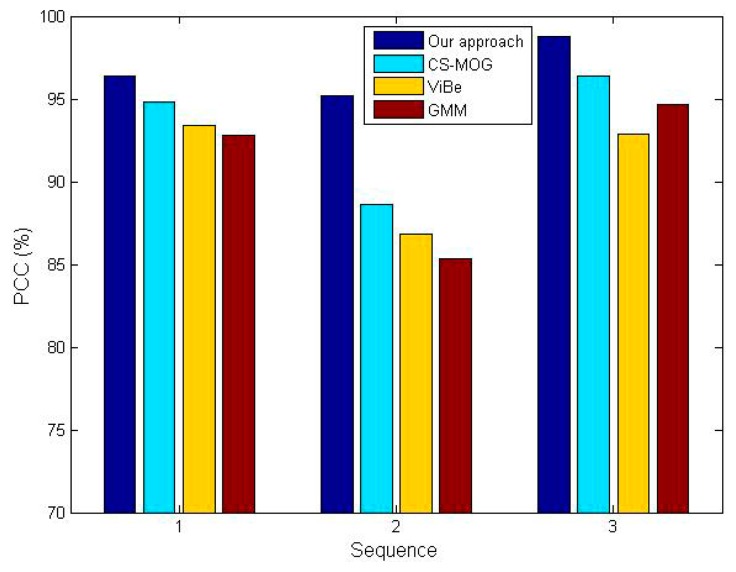
PCCs of four methods.

**Figure 12.**
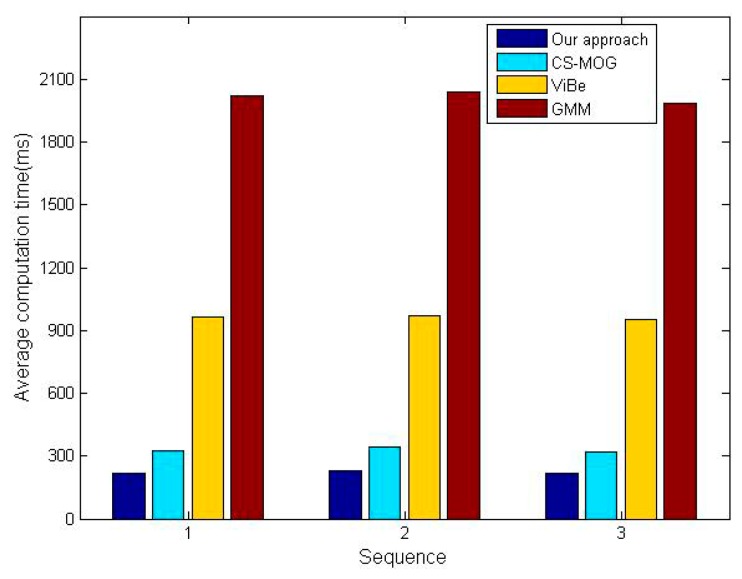
Average computation times (ms) of four methods.

Two metrics, the percentage of correct classification (PCC) [32] and average computation time, are adopted for the comparative tests. Note that the former needs to be as high as possible in order to minimize detection errors, instead, the latter should be as small as possible to save energy and hardware resources. The PCC scores of the four algorithms for three sequences are shown in Figure 11, which confirm our method gives the best performance in detection reliability. The PCC increments of our method vary from 6.6% to 10.3% in comparison with CS-MOG, ViBe and GMM. Furthermore, Figure 12 shows the average computation time per frame. We can observe that the methods using dimensionality reduction, our method and CS-MOG, are evidently faster than the pixel-based methods. For example, for the first sequence, the average computation times of our approach, CS-MOG, ViBe and GMM are 219.61, 324.47, 962.1 and 2018.87 ms, respectively. Besides, our approach is 32.3% faster than CS-MOG. From the foregoing, our approach performs best in terms of detection reliability and computation efficiency.

### 5.3. Realistic Deployment

The initial deployment aims to detect debris objects and collect pH and temperature data. This experiment is conducted in a pond located in our campus. A tiny scale aquatic sensor network consists of a host node (node 1), two slave nodes (node 5 and node 6) equipped with camera sensors, pH sensors and temperature sensors. The two slave nodes are submerged in the pond, and the host node is placed in our lab approximately 70 and 100 meters from the two slave nodes, respectively. Based on Z-Stack, the host node is configured as a coordinator, meanwhile, two slave nodes are configured as end-devices, and then these nodes form a star network. To initialize the network, the host node first sends out a broadcast signal containing its identity. Upon receiving the broadcast signal, the slave nodes remember the host node as their parent node and then send an acknowledgement back to indicate the communication link has been established. During monitoring, slave nodes transmit their data to the host node for processing and display.

**Figure 13.**
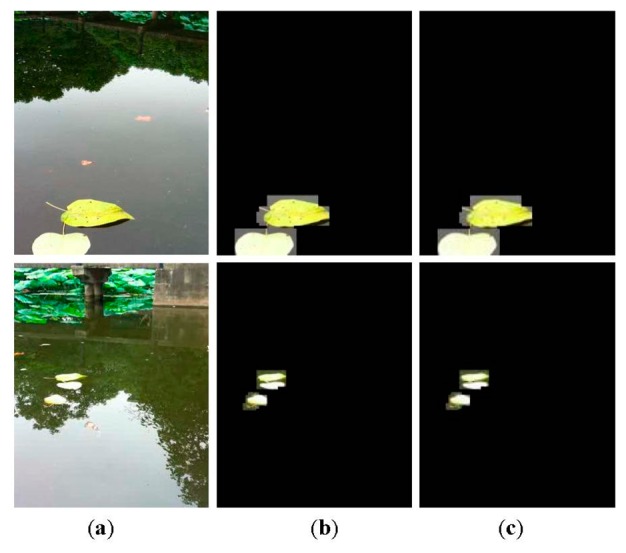
Debris detection (16 × 16 block size) using aquatic sensor nodes 5 and 6. (**a**) The images captured by two nodes; (**b**) The corresponding detection results; (**c**) The recovered images.

In our case, the sensing platform periodically sleeps for 30 s intervals and executes the debris detection algorithm after each wake-up. The camera frame rate is set to be 0.5 fps, nodes 5 and 6 perform the real-time, *in situ* debris detection in their respective field of view. Figure 13 shows the debris detection results of two aquatic sensor nodes, which validate the feasibility of the proposed method for embedded sensing platforms. It is worthwhile to note that the debris images in Figure 13c are recovered by the uploaded measurements according to Section 4.3, and the average normalized recovery error is 0.0201. At the end of a round of debris detection, the sensing platform chooses a desired value of block size for the next round based on the disturbance evaluation.

**Figure 14.**
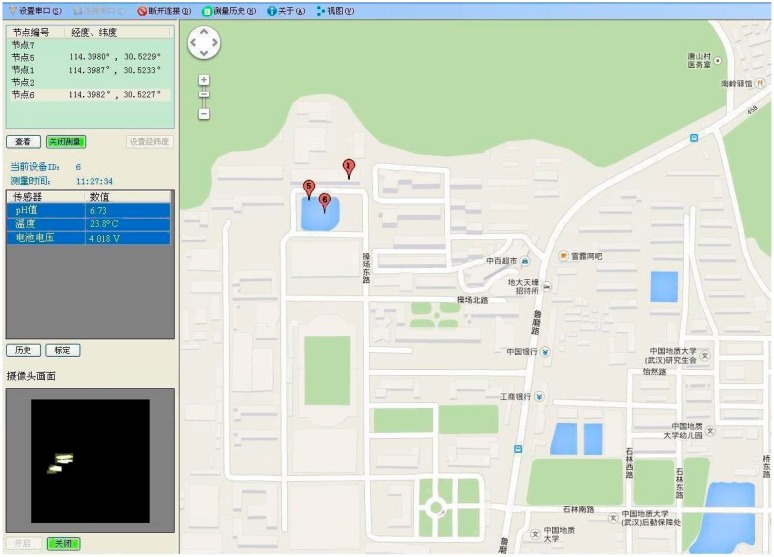
The user terminal screen of the host software.

Figure 14 illustrates the user terminal screen of the host software. Firstly, the software contains the functionality to call Google map in order to fix positions on the sensor nodes through GPS. We can see from the right section of the screen that the sites of the three nodes are clearly marked on the map. Secondly, the host software allows the user to choose sensor nodes, as well as displays the water-quality data on the computer screen. These data are collected once every 30 s and shown in a data window located in the middle of the left screen. The identity number of the selected node is above this window. As shown in Figure 14, the pH, temperature and battery voltage values measured by nodes 6, taken at 11:27 a.m., are 6.73, 23.8 °C and 4.018 V, respectively. Thirdly, the two buttons in the lower left corner allow the user to start and stop the camera sensor. When the camera sensor is activated, the debris detection will be executed. Once debris objects are detected, the measurements are uploaded to the user terminal. It can be seen that the debris image of node 6 is recovered and shown in an image window on the bottom left of the screen. Alternatively, the camera sensor can be switched off when not performing the debris detection.

Let's take node 6 for example to further analyze the efficiency of the sensing platform. At each interval, each of pH, temperature and battery voltage stores 2 bytes per reading. The GPS information contains 20 bytes per reading (*i.e.*, 10 bytes for longitude and 10 bytes for latitude). Besides, the measurements of the debris image require 10,260 bytes. This means that the total bytes needed to be transmitted are about 10 KB. Such a small payload size indicates that ZigBee radio with the data rate of 250 kbps has a low communication overhead and an extremely short latency. In the case of bad wireless network conditions, e.g., network congestion or intermittent connection, all the data records can be stored in onboard microSD card before they are uploaded. Specifically, an interval of 30 s has 2880 records each day, thus the total number of days is 145 days before the memory capacity is used up. Power consumption of sensor nodes is a critical issue that determines the lifetime of the network. After the preliminary test, the average current consumption is 290 mA when the sensing platform collects alone the water-quality data using aquatic sensors, where the test also utilizes ZigBee radio to transmit these data and GPS information. We then take the same measurement but in the meanwhile the debris detection based on camera sensor is enabled, the current consumption increases to 530 mA. As mentioned before, the sensing platform is powered by a 16,000 mAh battery. If the node works for 30 s every one minute, the battery of each sensor node can approximately last 60 h. Likewise, if the node only turns on for one minute every half an hour to collect sensor data and detect debris, the battery will last for 38 days on a full charge. As expected, the low power consumption makes our sensor nodes feasible for long-term debris monitoring.

## 6. Conclusions

In this paper, we first present an embedded sensing platform designed for aquatic environment monitoring. Based on this, we propose a lightweight debris detection algorithm, which effectively deals with environmental disturbances. The experiments demonstrate the feasibility and versatility of the proposed method in challenging environments. Moreover, real implementation on embedded sensing platforms shows that our method is more accurate, and consumes less hardware resources than the conventional approaches. Finally, an initial deployment of aquatic sensor nodes shows that the proposed method provides robust debris detection performance, meets the real-time requirement on embedded sensing platforms. Our future work will focus on the implementation of aquatic mobile platforms and collaboration schemes between multiple nodes for debris detection.
